# Characterization of Macrophage Phenotypes in Three Murine Models of House-Dust-Mite-Induced Asthma

**DOI:** 10.1155/2013/632049

**Published:** 2013-02-27

**Authors:** Christina Draijer, Patricia Robbe, Carian E. Boorsma, Machteld N. Hylkema, Barbro N. Melgert

**Affiliations:** ^1^Department of Pharmacokinetics, Toxicology and Targeting, Groningen Research Institute for Pharmacy, University of Groningen, Antonius Deusinglaan 1, 9713 AV Groningen, The Netherlands; ^2^GRIAC Research Institute, University Medical Centre Groningen, University of Groningen, Hanzeplein 1, 9713 GZ Groningen, The Netherlands; ^3^Department of Pathology, University Medical Center Groningen, University of Groningen, Hanzeplein 1, 9713 GZ Groningen, The Netherlands

## Abstract

In asthma, an important role for innate immunity is increasingly being recognized. Key innate immune cells in the lungs are macrophages. Depending on the signals they receive, macrophages can at least have an M1, M2, or M2-like phenotype. It is unknown how these macrophage phenotypes behave with regard to (the severity of) asthma. We have quantified the phenotypes in three models of house dust mite (HDM-)induced asthma (14, 21, and 24 days). M1, M2, and M2-like phenotypes were identified by interferon regulatory factor 5 (IRF5), YM1, and IL-10, respectively. We found higher percentages of eosinophils in HDM-exposed mice compared to control but no differences between HDM models. T cell numbers were higher after HDM exposure and were the highest in the 24-day HDM protocol. Higher numbers of M2 macrophages after HDM correlated with higher eosinophil numbers. In mice with less severe asthma, M1 macrophage numbers were higher and correlated negatively with M2 macrophages numbers. Lower numbers of M2-like macrophages were found after HDM exposure and these correlated negatively with M2 macrophages. The balance between macrophage phenotypes changes as the severity of allergic airway inflammation increases. Influencing this imbalanced relationship could be a novel approach to treat asthma.

## 1. Introduction

Asthma is characterized by irreversible obstruction and chronic inflammation of the airways, and is traditionally considered a T helper 2 (Th2-)cell driven inflammatory disorder [1]. However, an important role for the innate immune system in addition to the adaptive immune system is increasingly being recognized in asthma [2]. 

Macrophages are key cells in innate immune responses in the lung: they are among the most abundant cells and one of the first to encounter allergens and other threats to homeostasis. They also have the plasticity to quickly deal with those without endangering normal gas exchange. Depending on the signals received, macrophages can be pro- or anti-inflammatory, immunogenic or tolerogenic, and destroying or repairing tissue. Each characteristic may belong to a different macrophage phenotype with distinct functions [3, 4]. 

Tumor necrosis factor *α* (TNF*α*) and interferon *γ* (IFN*γ*) induce, under the influence of the transcription factor interferon-regulatory factor 5 (IRF5), a phenotype of M1 macrophages with increased microbicidal and/or tumoricidal activities [4, 5]. Exposure to IL-4 or IL-13 results in a population of M2 macrophages that is involved in anti-parasite and tissue repair responses [6, 7]. In mice, these cells are recognized by high production of chitinase and chitinase-like molecules such as YM1 [7, 8]. A close sibling of M2 macrophages is the M2-like macrophage phenotype. These macrophages can be induced by a variety of stimuli including exposure to a TLR-ligand in the presence of IL-10 or many more compounds. The main characteristic of the subtly different M2-like population is the production of IL-10. Since IL-10 is a potent anti-inflammatory cytokine, these M2-like macrophages are effective inhibitors of inflammation [4].

Despite the broad the spectrum of macrophage activation, the role of macrophages in asthma has scarcely been studied [9]. From what is known, all three macrophage phenotypes have been implicated in the development of murine and human asthma [10–12]. In mice, depending on the protocol used, asthma phenotypes can greatly differ [13, 14]. We aimed to investigate the distribution of the three main macrophages phenotypes in three different models of HDM-induced asthma and also included the effects of sex on asthma development. First, we show the general differences in airway inflammation in the three HDM models and next we study the distribution of the macrophage phenotypes with regard to severity of allergic airway inflammation. 

## 2. Materials and Methods

### 2.1. Animals

Male and female BALB/c mice (aged 6–8 weeks) were obtained from Harlan (Horst, The Netherlands). The mice were fed *ad libitum* with standard food and water and were held under specific pathogen-free conditions in groups of 4 mice per cage. The animal procedures, approved by the Institutional Animal Care and Use Committee of the University of Groningen (application number 5318), were performed under strict governmental and international guidelines. 

### 2.2. House Dust Mite (HDM)-Induced Airway Inflammation Models

Male (*n* = 4 per model) and female mice (*n* = 4 per model) were anaesthetized with isoflurane and exposed intranasally to whole body HDM extract (*Dermatophagoides pteronyssinus*, Greer laboratories, Lenoir, USA) in 40 *μ*L phosphate-buffered saline (PBS) according to three different protocols. Control animals (*n* = 8) were exposed to 40 *μ*L PBS according the 21-day protocol described. 

Mice of the first model (*n* = 8) received 100 *μ*g HDM extract intranasally on day 0, were subsequently exposed to 10 *μ*g HDM on day 7–11 according to the protocol of Hammad et al. and were sacrificed on day 14 (abbreviated as 14-day HDM) [15]. In the second model, according to Gregory et al. [16], mice (*n* = 8) were exposed to 25 *μ*g HDM extract three times a week during three weeks and were sacrificed on day 21 (abbreviated as 21-day HDM). For the last model (*n* = 7, due to illness one female was excluded from the study), mice were intranasally exposed to 100 *μ*g HDM on days 0, 7, 14 and 21 according to the protocol of Arora et al. and were sacrificed on day 24 (abbreviated as 24-day HDM) [17]. 

**Figure 1:**
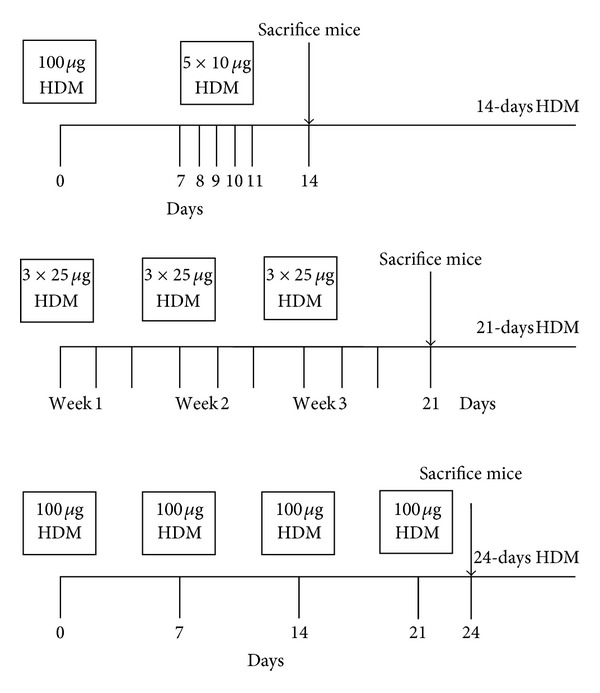
Experimental design of the study: three models of HDM-induced allergic inflammation. HDM, house dust mite.

During sacrifice lungs were lavaged three times with 1 mL cold PBS to determine the number of eosinophils and YM1 levels. Then, the left lung lobe was collected to isolate lung cells from digested lung for flow cytometry and the right lung was inflated with 0.5 mL 50% Tissue-Tek O.C.T. compound (Sakura, Finetek Europe B.V., Zoeterwoude, The Netherlands) in PBS and snap frozen/formalin-fixed for histological analyses. Serum was collected for analysis of HDM-specific IgE levels.  Figure 1 shows an overview of the experimental designs.

### 2.3. HDM-Specific IgE

Serum levels of HDM-specific IgE were measured by ELISA as described previously [18]. Arbitrary ELISA units of HDM-specific IgE titers were calculated as relative values to the optical density of pooled sera from HDM-exposed mice. 

### 2.4. Bronchoalveolar Lavage Fluid (BALF)

BALF was collected and total numbers of cells were determined using a Casy cell counter (Roche Innovatis AG, Reutlingen, Germany). After centrifugation at 300 ×gfor 10 minutes, BALF supernatants were stored at −80°C for further analysis (YM1 ELISA) and the cells were resuspended in RPMI medium (BioWhittaker Europe, Verviers, Belgium) for preparation of cytospots. Approximately 50,000 cells were spotted onto slides using a cytospin 3 (Thermo Shandon, Waltham, MA, USA) at 450 rpm for 5 minutes. To determine the percentage of eosinophils in the cytospots, a Giemsa staining (Sigma-Aldrich, Zwijndrecht, The Netherlands) was performed and the number of eosinophils was counted in a total of 300 cells.

The level of mECF-L (YM1) in BALF supernatants was determined by an ELISA kit according to the manufacturer's instructions (R&D Systems, Oxon, UK). 

### 2.5. Lung Digestion

After bronchoalveolar lavage, the left lung was minced and incubated in RPMI medium supplemented with 10% fetal calf serum (both Lonza, Verviers, Belgium), 10 *μ*g/mL DNAse I (grade II from bovine pancreas, Roche Applied Science, Almere, Netherlands), and 0.7 mg/mL collagenase A (Sigma-Aldrich) for 45 minutes at 37°C in a shaking water bath. The digested lung tissue was passed through a 70 *μ*m nylon strainer (BD Biosciences, Breda, Netherlands) to obtain single cell suspensions. Incubation with 10 times diluted Pharmlyse (BD Biosciences, Breda, The Netherlands) was performed to lyze contaminating erythrocytes. Cells were centrifuged through 70 *μ*m strainer caps and counted using a Casy cell counter (Roche Innovatis AG). Cells were subsequently used for flow cytometry.

### 2.6. Flow Cytometric Analysis

The single lung cell suspensions were stained for T-cell subsets using antibodies for flow cytometry. Frequencies of effector T cells (CD3+CD4+CD25+Foxp3−) and regulatory T cells (CD3+CD4+CD25+Foxp3+) were examined using *α*CD3-APC/Cy7 (Biolegend, Fell, Germany), *α*CD4-PE/Cy7 (Biolegend), *α*CD25-PE (Biolegend), and *α*Foxp3-APC (eBioscience, Vienna, Austria). An appropriate isotype control was used for the Foxp3 staining (rat IgG2ak-APC, eBioscience).

Approximately 10^6^ cells were incubated with the appropriate antibody mix including 1% normal mouse serum for 30 minutes on ice, protected from light. After washing the cells with PBS supplemented with 2% FCS and 5 mM EDTA, the cells were fixed and permeabilized for 30 minutes using a fixation and permeabilization buffer (eBioscience). Then cells were washed with permeabilization buffer and incubated with anti-Foxp3 including 1% normal mouse serum for 30 minutes. Subsequently, the cells were washed with permeabilization buffer, resuspended in FACS lysing solution (BD Biosciences) and kept in the dark on ice until flow cytometric analysis. The fluorescent staining of the cells was measured on a LSR-II flow cytometer (BD Biosciences) and data were analyzed using FlowJo Software (Tree Star, Ashland, USA).

### 2.7. Histology

Sections of 4 *μ*m were cut from the frozen part of the right lung and stained for all macrophages (rat *α*CD68, Serotec, Oxford, UK). The numbers of M2 macrophages were determined in frozen sections by staining for YM1 (goat *α*-mECF-L, R&D Systems, Oxon, UK) using standard immunohistochemical procedures. CD68 and YM1 were visualized with 3-amino-9-ethylcarbazole (AEC, Sigma Aldrich, Zwijndrecht, The Netherlands). 

The formalin-fixed part of the right lung was embedded in paraffinthen sections of 3 *μ*m were cut. To identify the M1 macrophages in tissue sections, antigen retrieval was performed by overnight incubation in Tris-HCL buffer pH 9.0 at 80°C and then sections were stained for IRF5 (rabbit *α*-IRF5, ProteinTech Europe, Manchester UK) using standard immunohistochemical procedures. To determine the number of IL-10 producing cells, antigen retrieval was performed by boiling the sections in citrate buffer pH 6.0 for 10 minutes. The sections were pretreated with 1% bovine serum albumin (Sigma Aldrich) and 5% milk powder in PBS for 30 minutes and incubated with rabbit *α*-IL-10 overnight (Hycult Biotech, Uden, The Netherlands). IRF5 and IL-10 were both visualized with ImmPACT NovaRED kit (Vector, Burlingame, CA, USA).

Positive cells were quantified by manual counting in parenchymal lung tissue (thus excluding large airways, vessels, and infiltrates, magnification 200–400x) and the total tissue area was quantified by morphometric analysis using ImageScope analysis software (Aperio, Vista, CA, USA). The numbers of cells were expressed per mm^2^ of tissue.

### 2.8. Statistical Analysis

To determine if the data were normally distributed a Kolmogorov-Smirnov test was used. If data sets were not normally distributed, appropriate transformations were performed. The differences between the models were tested using one-way analysis of variance (ANOVA) with Tukey's post-hoc test for multiple comparisons and sex differences were tested with the Student's *t* test. Pearson correlation coefficients were calculated to analyze the correlation between the inflammation parameters and macrophages phenotypes, and correlations within macrophage phenotypes (GraphPad Software, La Jolla, CA, USA). Differences were considered significant when *P* < 0.05, and *P* < 0.10 was considered a statistical trend.

## 3. Results

### 3.1. HDM Exposure Induces Allergic Airway Inflammation

To test whether exposure to HDM, according to three different protocols, induced allergic airway inflammation differently, we studied a number of general inflammation parameters.

**Figure 2:**
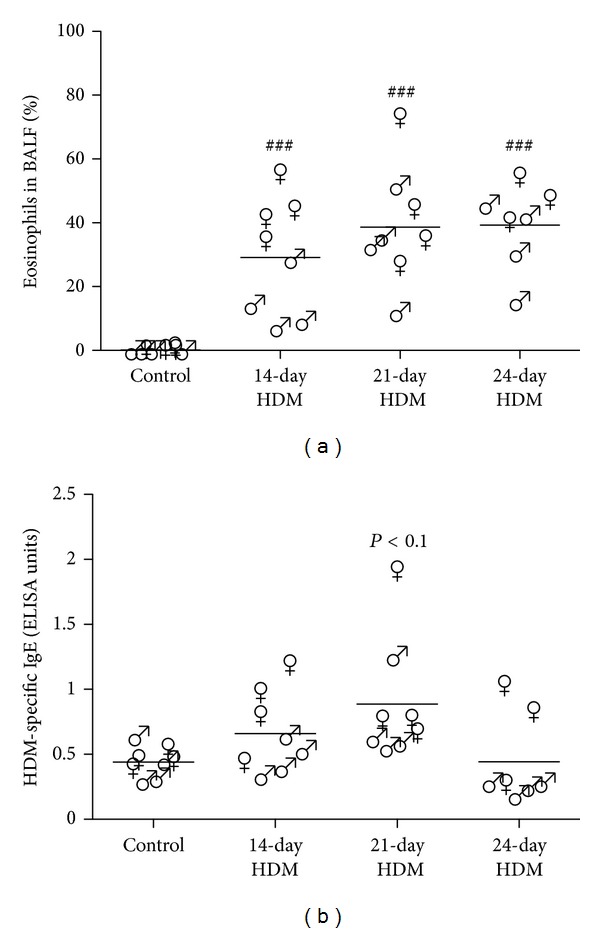
(a) HDM exposure induced higher percentages of eosinophils in BALF of males (♂) and females (♀) as compared to control, but no differences were found between the HDM protocols. Combining all models, higher percentages of eosinophils after HDM exposure were found in females as compared to males (*P* < 0.01). (b) The 21-day protocol increased HDM-specific IgE in serum of male and female mice as compared to control (trend, *P* < 0.1). Again combining all models, HDM exposure induced higher levels of HDM-specific IgE in females than in males (*P* < 0.05). ^ ###^ *P* < 0.001 compared to control.

Higher percentages of eosinophils in BALF were found in all three HDM-exposed groups as compared to control mice (Figure 2(a)). No differences in percentage of eosinophils in BALF were observed between the three HDM protocols. HDM-specific IgE levels in serum were not affected by the different HDM exposures, only a trend of higher levels was found in the group that was exposed to HDM in the 21-day protocol. In all protocols of HDM exposure, HDM-specific IgE levels were very low measuring just above the limit of detection in the calibration curve (Figure 2(b)). 

**Figure 3:**
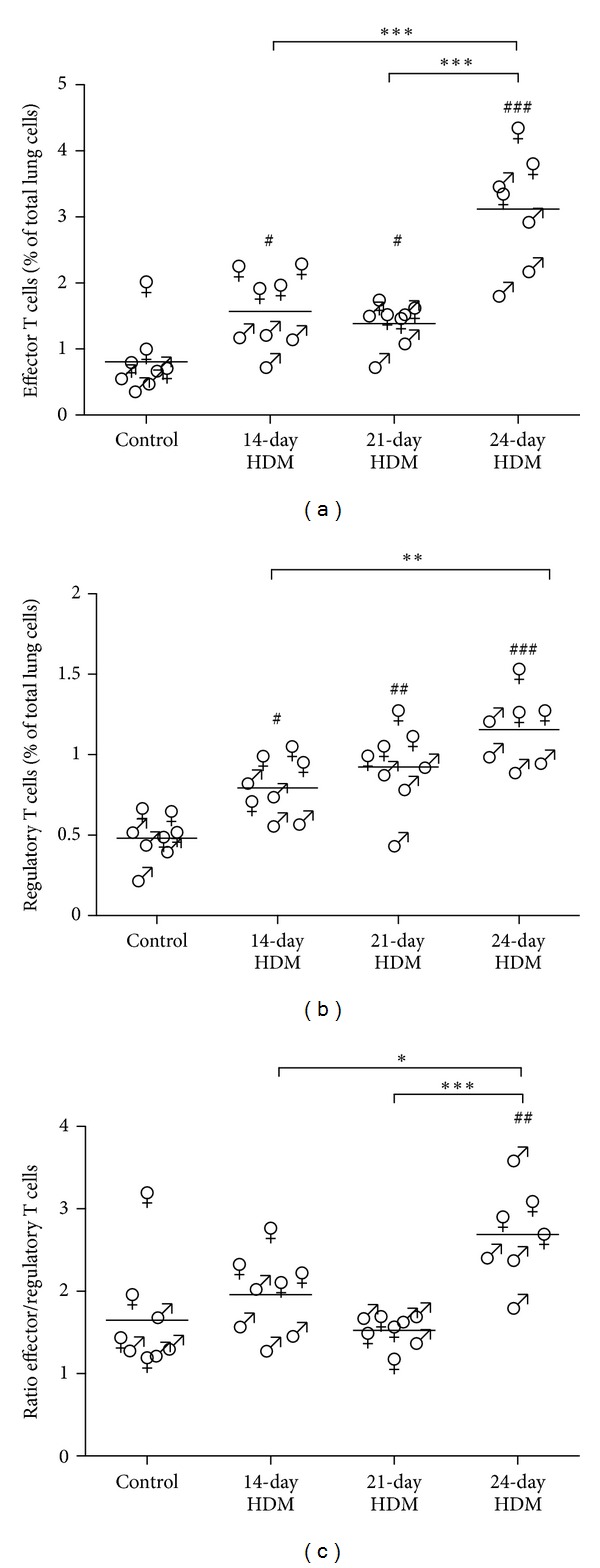
(a) HDM exposure induced higher percentages of effector T cells in lung tissue of males (♂) and females (♀) as compared to control. Higher percentages of effector T cells were found in the 24-day protocol as compared to the 14- and 21-day protocol of HDM exposure. Combining all models, HDM exposure induced higher percentages of effector T cells in females than in males (*P* < 0.05). (b) HDM exposure induced higher percentages of regulatory T cells in lung tissue of males and females as compared to control. Higher percentages of regulatory T cells were found in the 24-day protocol as compared to the 14-day protocol of HDM exposure. Combining all models, HDM exposure induced higher percentages of regulatory T cells in females than in males (*P* < 0.05). (c) The 24-day protocol had higher ratios of effector T cells to regulatory T cells in lung tissue of males and females as compared to control and the 14- and 21-day protocols of HDM exposure. Combining all models, no differences were found between males and females. ^#^ *P* < 0.05, ^##^ *P* < 0.01 and ^###^ *P* < 0.001 compared to control. *P < 0.05, **P < 0.01 and ***P < 0.001.

The higher airway inflammation in the three HDM-exposed groups was accompanied by higher percentages of effector T cells in lung tissue as compared to the control group (Figure 3(a)). The 24-day protocol showed a higher percentage of effector T cells in lungs than the 14- and 21-day protocol. After HDM exposure the percentage of regulatory T cells was also higher in all three protocols as compared to control mice (Figure 3(b)). The 24-day HDM protocol induced higher percentages of regulatory T cells in lungs compared to the 14-day protocol. The ratio of effector T cells to regulatory T cells was higher in the 24-day HDM protocol as compared to the control group and the other two HDM protocols (Figure 3(c)).

In females, HDM exposure induced more eosinophilia (*P* < 0.01), effector T cells (*P* < 0.05), regulatory T cells (*P* < 0.05), and higher levels of HDM-specific IgE (*P* < 0.05) than in males. 

### 3.2. HDM Exposure Induces M1 and M2 Macrophages but Inhibits M2-Like Macrophages

To study the presence of macrophage phenotypes after HDM exposure according to the three different protocols, we stained lung tissue for markers of total macrophages (CD68), M1 macrophages (IRF5), M2 macrophages (YM1), and M2-like macrophages (IL-10) and counted positive cells in parenchymal tissue. 

**Figure 4:**
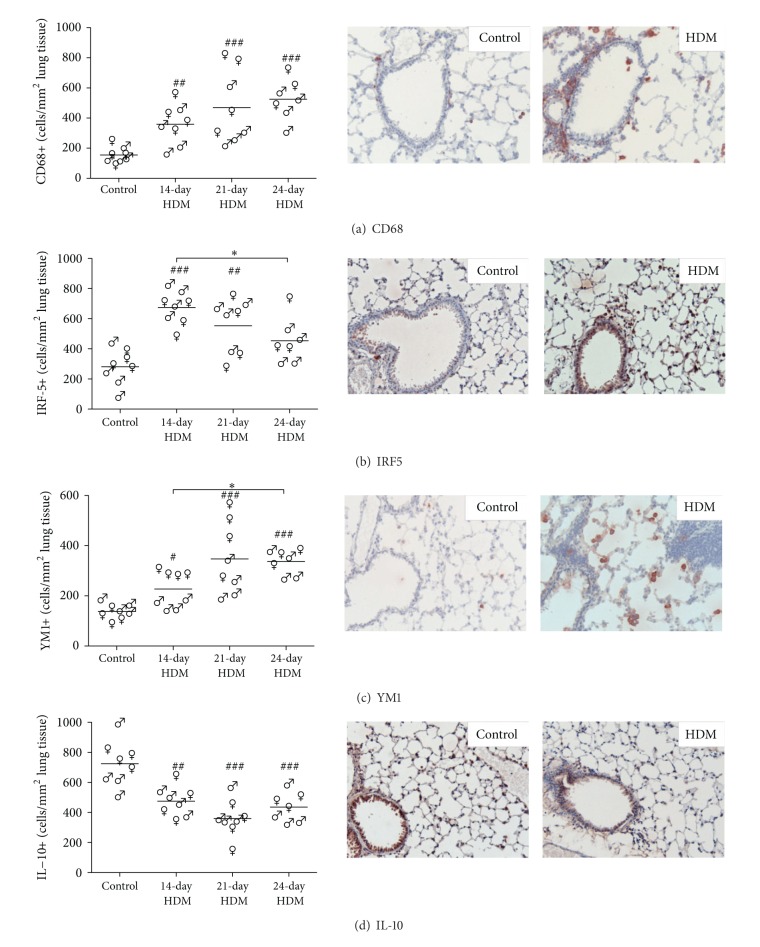
(a) HDM-exposed male (♂) and female (♀) mice had more CD68-positive cells in lung tissue as compared to control, but no differences were found between the HDM protocols. Combining all models, HDM exposure induced higher numbers of CD68-positive cells in females than in males (*P* < 0.05). (b) HDM-exposed male and female mice had more IRF5-positive cells in lung tissue as compared to control, but no differences were found between males and females when combining all models. The 14-day HDM protocol induced higher numbers of IRF5-positive cells as compared to the 24-day HDM protocol. (c) HDM-exposed male and female mice had more YM1-positive cells in lung tissue as compared to control, with higher numbers of YM1-positive cells in females than in males (*P* < 0.01). The 24-day HDM protocol induced higher numbers of YM1-positive cells as compared to the 14-day HDM protocol. (d) HDM-exposed male and female mice had lower numbers of IL-10-positive cells in lung tissue as compared to control, but no differences were found between the HDM protocols and between the sexes. The middle and right-hand panels are representative photos of control and HDM-exposed mice for all four stainings (magnification 200x). ^#^ *P* < 0.05, ^##^ *P* < 0.01 and ^###^ *P* < 0.001 compared to control. *P < 0.05.

HDM-exposed mice had more CD68-positive cells in lung tissue as compared to control mice (Figure 4(a)). No differences in CD68-positive numbers were observed between the HDM protocols. Compared to control mice, IRF5-positive cell numbers were higher in 14- and 21-day protocol, but not in mice exposed to HDM according to the 24-day protocol (Figure 4(b)). Between the HDM models, lower IRF5-positive numbers were found in lungs of mice that were exposed to HDM according to the 24-day protocol as compared to the mice of the 14-day HDM protocol. 

**Figure 5:**
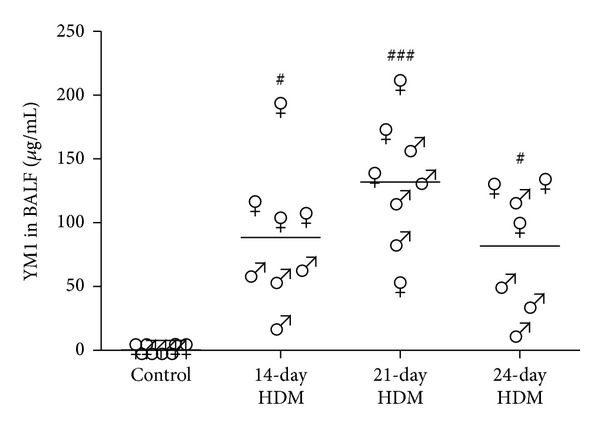
HDM exposure induced higher levels ofYM1 in bronchoalveolar lavage fluid (BALF) of male (♂) and female (♀) mice as compared to control, but no differences were found between the HDM protocols. Combining all models, HDM exposure induced higher levels of YM1 in BALF in females than in males (*P* < 0.05). ^#^ *P* = 0.05 and ^###^ *P* < 0.001 compared to control.

For YM1, all HDM protocols induced more YM1-positive cells as compared to control (Figure 4(c)). However, mice that were exposed in the 24-day HDM protocol had higher numbers of YM1-positive cells in lung tissue than the mice of the 14-day HDM protocol. YM1 levels in BALF were elevated in all HDM models as compared to control, but no differences were found between the models (Figure 5). Interestingly, HDM exposure resulted in significantly lower numbers of IL-10-positive cells in all three protocols compared to the control-treated group (Figure 4(d)). There were no differences observed in IL-10-positive cell numbers between the three HDM protocols. 

HDM-exposed females had more CD68-positive cells (*P* < 0.05), YM1-positive cells (*P* < 0.01), and higher levels of BALF YM1 (*P* < 0.05) than males, whereas no differences were found in IRF5- and IL-10-positve cells numbers between the two sexes.

### 3.3. M2 Macrophages Positively Correlate with Parameters of Airway Inflammation

To assess how severity of airway inflammation is reflected by the presence of the three main macrophage phenotypes, we correlated parameters of allergic airway inflammation with the different macrophage phenotypes in HDM-exposed mice (Table 1). 

**Figure 6:**
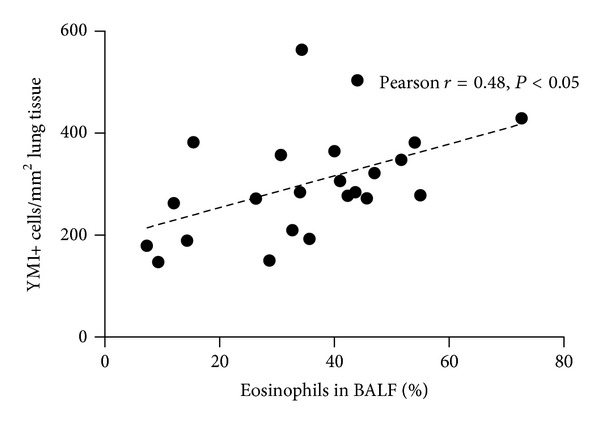
YM1-positive cell numbers (cells/mm^2^ lung tissue) correlated with percentage of eosinophils in BALF of male and female mice exposed to HDM according to three different protocols. No differences were found between males and females when combining all models.

Numbers of CD68-positive cells correlated positively with the percentage of eosinophils in BALF (*r* = 0.58), effector T cells (trend, *r* = 0.37) and regulatory T cells (*r* = 0.42) in lungs of HDM-exposed mice, indicating that more severe disease was accompanied by more macrophages. Most of these macrophages appear to be YM1-positive as only YM1-positive cell numbers correlated significantly with the percentage of eosinophils in BALF (*r* = 0.48, Figure 6) and the percentage of regulatory T cells (*r* = 0.51) in lung tissue. No differences were found between males and females. 

### 3.4. M2 Macrophages Negatively Correlate with IRF5-Positive and IL-10-Positive Cells

To study the relationship between the different macrophage phenotypes in allergic airway inflammation, correlations were made between YM1-postive, IRF5-positive, IL-10-positive, and CD68-positive cells in lung tissue of all HDM-exposed mice (Table 2).

**Figure 7:**
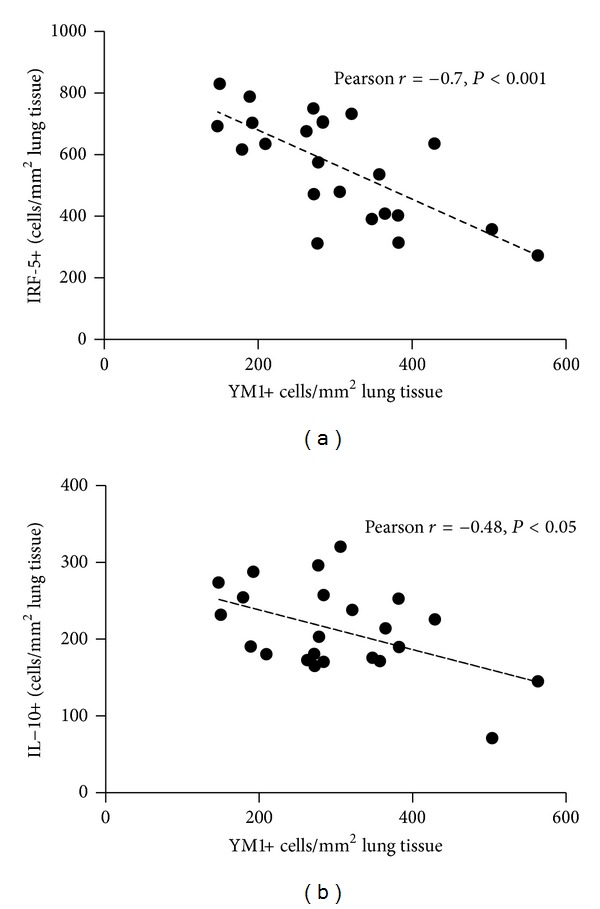
(a) YM1-positive cell numbers correlated with IRF5-positive cell numbers in lung tissue of male and female mice exposed to HDM according to 3 different protocols. (b) YM1-positive cells numbers correlated with IL-10-positive cell numbers in lung tissue of male and female mice exposed to HDM according to three different protocols. No differences were found between males and females when combining all models.

Numbers of IRF5-positive cells negatively correlated with cells positive for CD68 (trend, *r* = −0.40) and YM1 in lung tissue (*r* = −0.70, Figure 7(a)). YM1-positive cell numbers correlated negatively with numbers of IL-10-positve cells (*r* = −0.48, Figure 7(b)) and positively with numbers of CD68-positive cells in lung tissue (*r* = 0.66). No differences were found between males and females. 

## 4. Discussion

Our study has shown that the balance between macrophage phenotype changes as the severity of allergic inflammation increases. Higher numbers of M2 macrophages in HDM-exposed mice correlated with higher percentages of eosinophils in BALF. At the same time lower numbers of M1 macrophages and M2-like macrophages were found in the mice with more severe inflammation and these therefore correlated negatively with M2 macrophages. In addition, we have confirmed again that females have more pronounced airway inflammation with higher numbers of M2 macrophages as compared to males [19].

The models we used for our study were short-term exposure to HDM and they give us much information about the distribution of the different macrophage phenotypes during induction of asthma. In these models we found that longer exposure to HDM did not induce more severe eosinophilic inflammation but it did lead to higher numbers of M2 macrophages and higher percentages of effector and regulatory T cells in lungs of mice. The fact that we found no differences in eosinophils between the models is probably due to the large variation within the groups. However, when analyzing all HDM-exposed mice separately, eosinophils correlated positively with total macrophages and M2 macrophages, confirming our previous findings in humans that M2 macrophages increase with increasing asthma severity [20]. Another important parameter of allergic airway inflammation, serum HDM-specific IgE, could barely be detected probably because the duration of the models was too short. It takes around 3 weeks for naive B cells to mature to plasma cells and switch from IgM production to IgE after first contact with an antigen [21]. Our models lasted 24 days at the most and we therefore sacrificed our animals before a full-blown IgE response could develop. Also, studies from other groups using these models did not show HDM-specific IgE in serum [15–17]. To investigate how macrophage phenotypes are distributed during and contribute to more chronic disease, longer models of HDM-exposure need to be used.

We sought to phenotype the distinct macrophage subsets in lung tissue using markers that could distinguish each phenotype. We identified M2 macrophages using expression of YM1, which is unique for this phenotype in the lung. Previous studies found that only macrophages express YM1 and the staining is not complicated by other cells staining positive [22–24]. A unique marker for identifying M1 macrophages in lung tissue is, however, more difficult to find. To our knowledge selective surface markers for tissue are not available and therefore the production of IL-12 and oxygen radicals have been used in several mouse studies [25, 26]. In asthma, oxygen radicals cannot be used to stain for M1 macrophages because these are also copiously produced by eosinophils that are present in great numbers [27]. Recently, IRF5 was found to be the transcription factor controlling M1 differentiation. It is highly expressed in M1 macrophages while it suppresses the M2 phenotype [5]. Our study was the first to use IRF5 as an M1 marker on lung tissue and it is a fairly selective marker. Bronchial epithelial cells and incoming leukocytes in infiltrates also stain positive for IRF5 but these could be excluded from the quantification of parenchymal tissue based on localization. A double staining with CD68 to make sure only macrophages are included in the quantification is unfortunately at present not possible due to technical incompatibilities. In previous studies, M2-like macrophages are often not distinguished from M2 macrophages because they share many markers, including mannose receptors. The production of the immunosuppressive cytokine IL-10 is the most important and reliable characteristic of M2-like macrophages and can be used to identify these cells with [4]. Similar to IRF5, bronchial epithelial cells and some cells in infiltrates are also expressing IL-10 but these can easily be excluded from the quantification of parenchymal tissue. To exclude other IL-10-producing cells from the analysis a double staining with CD68 is needed, but at present also not possible. These limitations in the stainings for IRF5 and IL-10 may also explain why the total number of YM1-, IRF5- and IL-10-positive cells is higher than the number of CD68-positive cells.

Higher numbers of M2 macrophages were found in lungs of HDM-exposed mice, which correlates with previous studies [19, 20, 28]. Interestingly, numbers of M2 macrophages and the levels of YM1 in BALF correlated strongly with eosinophils in BALF. YM1 is also known as eosinophil chemotactic factor (ECF-L) [29], but it was suggested that YM1 only has weak chemotactic properties for eosinophils [24, 30]. The chemotactic strength of YM1 is still debated and our findings and those of others do suggest otherwise [23, 31]. The number of M2 macrophages also showed a positive correlation with regulatory T cells. In an interesting study by Tiemessen et al., regulatory T cells were shown to promote the induction of M2 macrophages to help with trying to maintain tissue homeostasis and preventing too much tissue damage of the inflammatory response [32]. Our data could be explained along these lines with incoming regulatory T cells inducing M2 macrophages in an attempt to restrict inflammation and tissue damage induced by HDM. The M2 macrophages would then be the result of inflammation and be beneficial instead of contributing to allergic airway inflammation, which is still an ongoing debate [19, 33, 34]. 

Asthma is dominated by a Th2-driven inflammation, but evidence shows that M1 macrophages are also present in this disease. In two interesting studies, IFN*γ*-stimulated macrophages were shown to prevent the onset of allergic airway inflammation [35, 36]. Our findings with higher numbers of M1 macrophages in the shorter protocols and in less severe diseases suggest that these macrophages are induced as a counterregulatory mechanism to dampen inflammation. However, higher numbers of M1 macrophages in asthma have also been shown in severe asthma and markers of M1 macrophages correlate with asthma severity, suggesting that M1 macrophages play a role in severe asthma as well [37–39]. This appears not to be the case for our results because we find higher numbers of M1 macrophages at the onset of allergic airway inflammation when numbers of effector T cells are lower, and lower numbers of M1 macrophages in the 24-day HDM protocol when effector T-cell numbers are higher. M1 macrophages appear to have a beneficial role in preventing allergic sensitization, but in already established disease they promote the development of a severe phenotype. A similar double role has been reported for the M1 cytokine IL-12 in allergic airway inflammation [40]. Since our HDM models are short and focus on the onset of the disease, we do not have information on the presence of M1 macrophages in established, more severe disease. 

Since M2-like macrophages have anti-inflammatory functions, we were interested in the presence of this macrophage phenotype in HDM-induced asthma. Others have reported that interstitial macrophages are the IL-10-producing macrophages and treatment of sensitized mice with these macrophages prevented the development of allergic airway inflammation [41]. Our observation of lower numbers of M2-like macrophages in asthma as compared to control is in line with these and previous findings in human asthmatics [42, 43]. Interestingly, it was shown that IL-10 production in severe asthmatics is even lower as compared to moderate asthmatics and that treatment with corticosteroids induces IL-10 production by macrophages [43, 44]. This suggests that specific stimulation of macrophages to polarize to M2-like macrophages that produce IL-10 may reduce asthma symptoms. 

Women suffer from more severe asthma than men and this phenomenon was also found in mouse models of asthma [45, 46]. A role for sex hormones has been suggested, but the underlying mechanisms are unknown [47]. In accordance with the previous findings, we show that HDM-exposed female mice had more pronounced airway inflammation than male mice. In macrophage phenotypes, we only found a difference in the M2 macrophages. Since we and others found a correlation between M2 macrophages and asthma severity, it may suggest that this phenotype could play a role in the increased airway inflammation in females [19].

## 5. Conclusion

Taken together, our data suggest that during the development of allergic airway inflammation M1 and M2 macrophages are induced or recruited, whereas M2-like macrophages are prevented from moving in or inhibited. As HDM-induced inflammation progresses, M1 macrophages are diminished in favor of M2 macrophages possibly under the influence of regulatory T cells that try to restrict inflammation. Their work may be hampered by the fact that IL-10-producing M2-like macrophages do not develop in HDM-induced inflammation and the inflammation can progress. Influencing this imbalanced relationship by therapeutic macrophage targeting could be a novel way to treat asthma.

## Figures and Tables

**Table 1:** Correlations between macrophage phenotype markers and parameters of allergic airway inflammation.

	CD68+ cells	IRF5+ cells	YM1+ cells	IL-10+ cells
Eosinophils	0.58**	−0.26	0.48*	0.01
HDM-specific IgE	−0.06	−0.18	0.33	−0.13
Effector T cells	0.37^#^	−0.30	0.29	0.05
Regulatory T cells	0.42*	−0.20	0.51*	−0.21

Values are correlations coefficients (Pearson correlation). ^#^ *P* < 0.1, *P < 0.05 and **P < 0.01.

**Table 2:** Correlations between macrophage phenotype markers.

	CD68+ cells	IRF5+ cells	YM1+ cells	IL-10+ cells
CD68+ cells		−0.40^#^	0.66**	−0.17
IRF5+ cells			−0.70**	0.20
YM1+ cells				−0.48*

Values are correlations coefficients (Pearson correlation). ^#^ *P* < 0.1, *P < 0.05 and **P < 0.01.

## References

[1] Barnes PJ (2008). Immunology of asthma and chronic obstructive pulmonary disease. *Nature Reviews Immunology*.

[2] Anderson GP (2008). Endotyping asthma: new insights into key pathogenic mechanisms in a complex, heterogeneous disease. *The Lancet*.

[3] Stout RD, Suttles J (2004). Functional plasticity of macrophages: reversible adaptation to changing microenvironments. *Journal of Leukocyte Biology*.

[4] Mosser DM, Edwards JP (2008). Exploring the full spectrum of macrophage activation. *Nature Reviews Immunology*.

[5] Krausgruber T, Blazek K, Smallie T (2011). IRF5 promotes inflammatory macrophage polarization and T H1-TH17 responses. *Nature Immunology*.

[6] Kreider T, Anthony RM, Urban Jr JF, Gause WC (2007). Alternatively activated macrophages in helminth infections. *Current Opinion in Immunology*.

[7] Martinez FO, Helming L, Gordon S (2009). Alternative activation of macrophages: an immunologic functional perspective. *Annual Reviews*.

[8] Nair MG, Cochrane DW, Allen JE (2003). Macrophages in chronic type 2 inflammation have a novel phenotype characterized by the abundant expression of Ym1 and Fizz1 that can be partly replicated in vitro. *Immunology Letters*.

[9] Peters-Golden M (2004). The alveolar macrophage: the forgotten cell in asthma. *American Journal of Respiratory Cell and Molecular Biology*.

[10] Moreira AP, Hogaboam CM (2011). Macrophages in allergic asthma: fine-tuning their pro- and anti-inflammatory actions for disease resolution. *Journal of Interferon and Cytokine Research*.

[11] Dasgupta P, Keegan AD (2012). Contribution of alternatively activated macrophages to allergic lung Inflammation: a tale of mice and men. *Journal of Innate Immunity*.

[12] Yang M, Kumar RK, Hansbro PM, Foster PS (2012). Emerging roles of pulmonary macrophages in driving the development of severe asthma. *Journal of Leukocyte Biology*.

[13] Chang YS, Kim YK, Bahn JW (2005). Comparison of asthma phenotypes using different sensitizing protocols in mice. *Korean Journal of Internal Medicine*.

[14] Nials AT, Uddin S (2008). Mouse models of allergic asthma: acute and chronic allergen challenge. *Disease Models and Mechanisms*.

[15] Hammad H, Plantinga M, Deswarte K (2010). Inflammatory dendritic cells—not basophils—are necessary and sufficient for induction of Th2 immunity to inhaled house dust mite allergen. *The Journal of Experimental Medicine*.

[16] Gregory LG, Mathie SA, Walker SA, Pegorier S, Jones CP, Lloyd CM (2010). Overexpression of Smad2 drives house dust mite-mediated airway remodeling and airway hyperresponsiveness via activin and IL-25. *American Journal of Respiratory and Critical Care Medicine*.

[17] Arora M, Poe SL, Oriss TB (2010). TLR4/MyD88-induced CD11b^+^Gr − 1^int^F4/80^+^ non-migratory myeloid cells suppress Th2 effector function in the lung. *Mucosal Immunology*.

[18] Blacquière MJ, Timens W, Melgert BN, Geerlings M, Postma DS, Hylkema MN (2009). Maternal smoking during pregnancy induces airway remodelling in mice offspring. *European Respiratory Journal*.

[19] Melgert BN, Oriss TB, Qi Z (2010). Macrophages: regulators of sex differences in asthma?. *American Journal of Respiratory Cell and Molecular Biology*.

[20] Melgert BN, Ten Hacken NH, Rutgers B, Timens W, Postma DS, Hylkema MN (2011). More alternative activation of macrophages in lungs of asthmatic patients. *Journal of Allergy and Clinical Immunology*.

[21] Abbas AK, Lichtman AH, Pillai S (2011). *Cellular and Molecular Immunology*.

[22] Raes G, de Baetselier P, Noël W, Beschin A, Brombacher F, Gholamreza HG (2002). Differential expression of FIZZ1 and Ym1 in alternatively versus classically activated macrophages. *Journal of Leukocyte Biology*.

[23] Loke P, Nair MG, Parkinson J, Guiliano D, Blaxter M, Allen JE (2002). IL-4 dependent alternatively-activated macrophages have a distinctive in vivo gene expression phenotype. *BMC Immunology*.

[24] Welch JS, Escoubet-Lozach L, Sykes DB, Liddiard K, Greaves DR, Glass CK (2002). TH2 cytokines and allergic challenge induce Ym1 expression in macrophages by a STAT6-dependent mechanism. *Journal of Biological Chemistry*.

[25] Bastos KRB, Alvarez JM, Marinho CRF, Rizzo LV, Lima MRD (2002). Macrophages from IL-12p40-deficient mice have a bias toward the M2 activation profile. *Journal of Leukocyte Biology*.

[26] Sindrilaru A, Peters T, Wieschalka S (2011). An unrestrained proinflammatory M1 macrophage population induced by iron impairs wound healing in humans and mice. *Journal of Clinical Investigation*.

[27] Nagata M (2005). Inflammatory cells and oxygen radicals. *Current Drug Targets*.

[28] Chupp GL, Lee CG, Jarjour N (2007). A chitinase-like protein in the lung and circulation of patients with severe asthma. *The New England Journal of Medicine*.

[29] Owhashi M, Arita H, Hayai N (2000). Identification of a novel eosinophil chemotactic cytokine (ECF-L) as a chitinase family protein. *Journal of Biological Chemistry*.

[30] Chang NCA, Hung SI, Hwa KY (2001). A macrophage protein, Ym1, transiently expressed during inflammation is a novel mammalian lectin. *Journal of Biological Chemistry*.

[31] Voehringer D, van Rooijen N, Locksley RM (2007). Eosinophils develop in distinct stages and are recruited to peripheral sites by alternatively activated macrophages. *Journal of Leukocyte Biology*.

[32] Tiemessen MM, Jagger AL, Evans HG, van Herwijnen MJC, John S, Taams LS (2007). CD4^+^CD25^+^Foxp3^+^ regulatory T cells induce alternative activation of human monocytes/macrophages. *Proceedings of the National Academy of Sciences of the United States of America*.

[33] Ford AQ, Dasgupta P, Mikhailenko I, Smith EMP, Noben-Trauth N, Keegan AD (2012). Adoptive transfer of IL-4R*α*
^+^ macrophages is sufficient to enhance eosinophilic inflammation in a mouse model of allergic lung inflammation. *BMC Immunology*.

[34] Nieuwenhuizen NE, Kirstein F, Jayakumar J (2012). Allergic airway disease is unaffected by the absence of IL-4Ra-dependent alternatively activated macrophages. *The Journal of Allergy and Clinical Immunology*.

[35] Korf JE, Pynaert G, Tournoy K (2006). Macrophage reprogramming by mycolic acid promotes a tolerogenic response in experimental asthma. *American Journal of Respiratory and Critical Care Medicine*.

[36] Tang C, Inman MD, van Rooijen N (2001). Th type 1-stimulating activity of lung macrophages inhibits Th2-mediated allergic airway inflammation by an IFN-*γ*-dependent mechanism. *Journal of Immunology*.

[37] Goleva E, Hauk PJ, Hall CF (2008). Corticosteroid-resistant asthma is associated with classical antimicrobial activation of airway macrophages. *Journal of Allergy and Clinical Immunology*.

[38] Ten Hacken NHT, Oosterhoff Y, Kauffman HF (1998). Elevated serum interferon-*γ* in atopic asthma correlates with increased airways responsiveness and circadian peak expiratory flow variation. *European Respiratory Journal*.

[39] Wang C, Rose-Zerilli MJ, Koppelman GH (2012). Evidence of association between interferon regulatory factor 5 polymorphisms and asthma. *Gene Gene*.

[40] Meyts I, Hellings PW, Hens G (2006). IL-12 contributes to allergen-induced airway inflammation in experimental asthma. *Journal of Immunology*.

[41] Bedoret D, Wallemacq H, Marichal T (2009). Lung interstitial macrophages alter dendritic cell functions to prevent airway allergy in mice. *Journal of Clinical Investigation*.

[42] Maneechotesuwan K, Supawita S, Kasetsinsombat K, Wongkajornsilp A, Barnes PJ (2008). Sputum indoleamine-2, 3-dioxygenase activity is increased in asthmatic airways by using inhaled corticosteroids. *Journal of Allergy and Clinical Immunology*.

[43] John M, Lim S, Seybold J (1998). Inhaled corticosteroids increase interleukin-10 but reduce macrophage inflammatory protein-1*α*, granulocyte-macrophage colony-stimulating factor, and interferon-*γ* release from alveolar macrophages in asthma. *American Journal of Respiratory and Critical Care Medicine*.

[44] Fitzpatrick AM, Higgins M, Holguin F, Brown LAS, Teague WG (2010). The molecular phenotype of severe asthma in children. *Journal of Allergy and Clinical Immunology*.

[45] Almqvist C, Worm M, Leynaert B (2008). Impact of gender on asthma in childhood and adolescence: a GA 2LEN review. *Allergy*.

[46] Melgert BN, Postma DS, Kuipers I (2005). Female mice are more susceptible to the development of allergic airway inflammation than male mice. *Clinical and Experimental Allergy*.

[47] Melgert BN, Ray A, Hylkema MN, Timens W, Postma DS (2007). Are there reasons why adult asthma is more common in females?. *Current Allergy and Asthma Reports*.

